# Multi-dimensional evaluation of cardiotoxicity in mice following respiratory exposure to polystyrene nanoplastics

**DOI:** 10.1186/s12989-023-00557-3

**Published:** 2023-11-29

**Authors:** Tianyi Zhang, Sheng Yang, Yiling Ge, Xin Wan, Yuxin Zhu, Fei Yang, Jie Li, Saisai Gong, Yanping Cheng, Chengyu Hu, Zaozao Chen, Lihong Yin, Yuepu Pu, Geyu Liang

**Affiliations:** 1 Key Laboratory of Environmental Medicine Engineering, Ministry of Education, School of Public Health, Southeast University, Nanjing, 210009 Jiangsu China; 2 Hunan Province Key Laboratory of Typical Environmental Pollution and Health Hazards, School of Public Health, Hengyang Medical School, University of South China, Hengyang, 421001 China; 3 State Key Laboratory of Bioelectronics, School of Biological Science and Medical Engineering, Southeast University, Nanjing, 210096 Jiangsu China

**Keywords:** PS-NPs, Respiratory exposure, Cardiotoxicity, High-throughput sequencing

## Abstract

**Background:**

Nanoplastics (NPs) could be released into environment through the degradation of plastic products, and their content in the air cannot be ignored. To date, no studies have focused on the cardiac injury effects and underlying mechanisms induced by respiratory exposure to NPs.

**Results:**

Here, we systematically investigated the cardiotoxicity of 40 nm polystyrene nanoplastics (PS-NPs) in mice exposed via inhalation. Four exposure concentrations (0 µg/day, 16 µg/day, 40 µg/day and 100 µg/day) and three exposure durations (1 week, 4 weeks, 12 weeks) were set for more comprehensive information and RNA-seq was performed to reveal the potential mechanisms of cardiotoxicity after acute, subacute and subchronic exposure. PS-NPs induced cardiac injury in a dose-dependent and time-dependent manner. Acute, subacute and subchronic exposure increased the levels of injury biomarkers and inflammation and disturbed the equilibrium between oxidase and antioxidase activity. Subacute and subchronic exposure dampened the cardiac systolic function and contributed to structural and ultrastructural damage in heart. Mechanistically, violent inflammatory and immune responses were evoked after acute exposure. Moreover, disturbed energy metabolism, especially the TCA cycle, in the myocardium caused by mitochondria damage may be the latent mechanism of PS-NPs-induced cardiac injury after subacute and subchronic exposure.

**Conclusion:**

The present study evaluated the cardiotoxicity induced by respiratory exposure to PS-NPs from multiple dimensions, including the accumulation of PS-NPs, cardiac functional assessment, histology observation, biomarkers detection and transcriptomic study. PS-NPs resulted in cardiac injury structurally and functionally in a dose-dependent and time-dependent manner, and mitochondria damage of myocardium induced by PS-NPs may be the potential mechanism for its cardiotoxicity.

**Graphical abstract:**

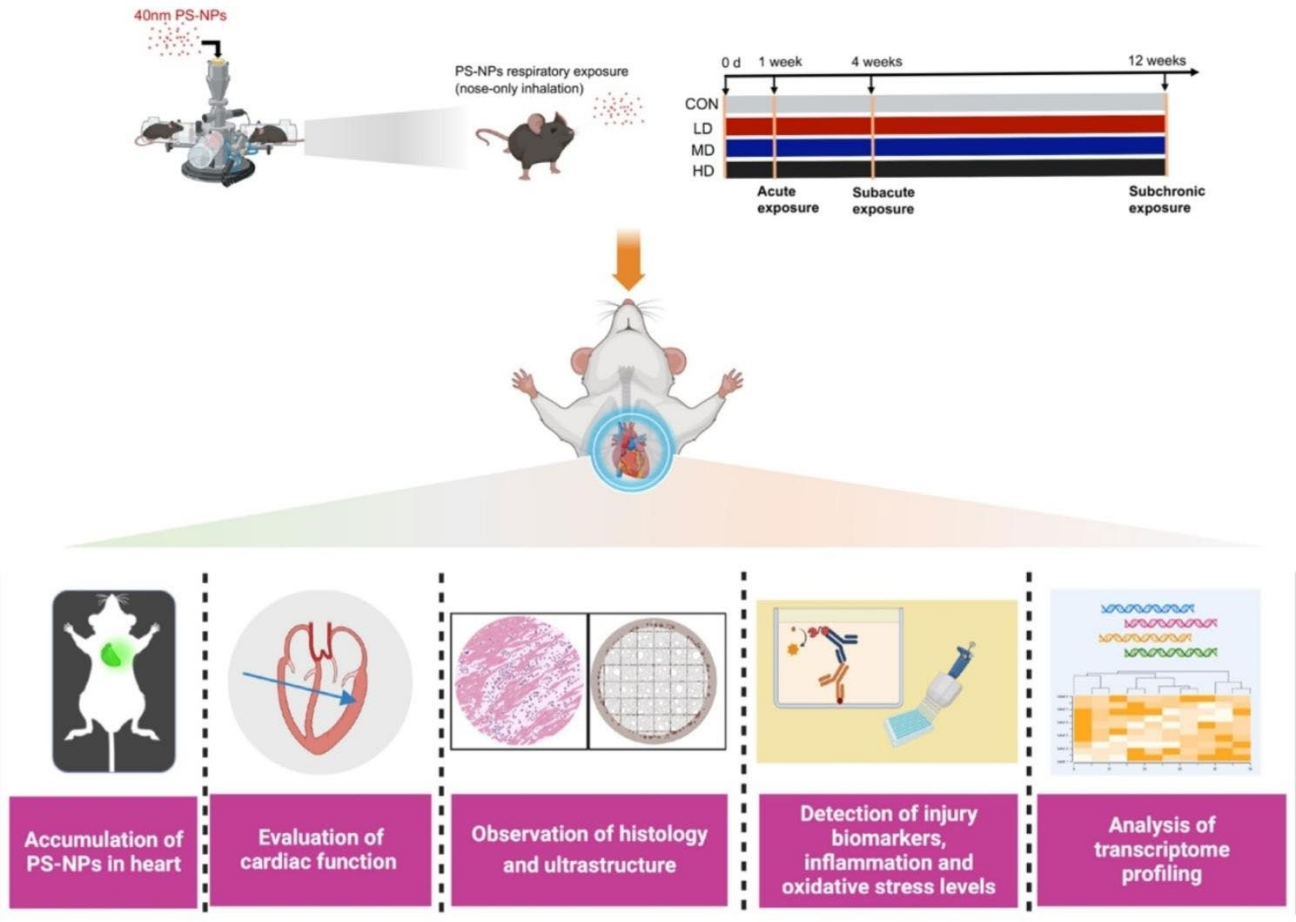

**Supplementary Information:**

The online version contains supplementary material available at 10.1186/s12989-023-00557-3.

## **Background**

“White pollution”, which is caused by plastic waste, has already been a hard nut to crack all over the world [1–3]. Moreover, plastic products remaining in the environment decompose into microplastics (MPs) (< 5 mm) and nanoplastics (NPs) (< 100 nm) under physical, chemical and biological processes, posing a huge threat to human health [4]. As a new type of pollutant, the migration and destiny of micro(nano) plastics (MNPs) in the environment have garnered significant interest. The majority of current research focuses on the health hazards caused by the intake of MNPs derived from drinking water and aquatic organisms [5, 6]. However, with the improvement of detection technology and the deepening of research, it has been found that the amount of MNPs in the air environment cannot be ignored. MNPs from indoor and outdoor sources such as clothing fibers, decorative materials, 3D printing, automobile tire wear, garbage incineration and plastic particles in urban dust are easily suspended in the air, leading to a dramatic increase in their concentration in the air [7]. In addition, due to their small particle size and light weight, MNPs in seawater bubbles are easily released into the air after they are broken and follow the updraft into the troposphere of the atmosphere, bringing about intercontinental and transoceanic transportation and then migrating around the world [8, 9]. Moreover, the mass production and use of protective materials, such as masks, have greatly exacerbated respiratory exposure to MNPs during the COVID-19 pandemic [10]. According to recent research data, 224 particles/m^3^ can be detected in outdoor air in Wenzhou, China, and the indoor concentration is as high as 1583 particles/m^3^. Researchers estimated that each Wenzhou person would inhale 1 million MNPs particles from outdoor air every year [11]. A much bigger concern is that considerable amounts of MNPs have been detected in both human lung tissues and bronchoalveolar lavage fluid (BALF) according to population evidence [12, 13]. Therefore, the respiratory tract is an important way for the human body to be exposed to MNPs, and it is urgent to carry out in-depth research on their toxic effects and mechanisms.

MNPs have been found to be prone to enter the blood system and accumulate in multiple organs, causing a broad range of toxicological effects, e.g., hepatotoxicity [14], neurotoxicity [15] and reproductive toxicity [16]. Moreover, a growing body of studies has demonstrated that the heart is also the target for MNPs accumulation and that exposure to MNPs is a new cardiovascular risk factor [17]. It has been reported that ingested MNPs could aggregate in the hearts of a variety of model animals, such as zebrafish, Atlantic horse mackerel, Wistar rats, and C57BL/6J mice, and cause multiple cardiac damage effects [18]. Anastasia et al. showed that MPs treatment significantly reduced zebrafish cardiac contraction frequency and increased cardiac oxidative stress levels [19]. Zhang et al. found that oral exposure to polystyrene nanoplastics (PS-NPs) for 42 days caused severe pathological damage and ultrastructural changes in chicken hearts, with aggravated inflammatory cell infiltration, mitochondria damage and cardiomyocyte apoptosis [20]. Additionally, the results of Li et al. also showed that intake of PS-NPs by drinking water for 90 days induced an increase in the level of oxidative stress in rat cardiomyocytes and promoted cardiac fibrosis [21].

However, to the best of our knowledge, all current studies on MNPs-induced cardiotoxicity are limited to oral exposure, and little is known about potential cardiac injury via the respiratory route. In addition, the selected particle sizes and exposure periods vary dramatically in different studies, and it is difficult to compare the cardiovascular toxicity effects horizontally or vertically. Moreover, in most studies, zebrafish have been widely used to explore the cardiotoxicity induced by MNPs, whereas experiments on mammalian models whose hearts are more similar to that of human body in structure and function need to be further performed to study the phenotypes and mechanisms.

To fill the gaps in existing research, we chose PS-NPs, which is one of the most ubiquitous NPs in the environment [22, 23], as the representative pollutant to carry out respiratory exposure experiments in mice. Three exposure dosages, including low dose (LD), medium dose (MD) and high dose (HD), were designed to investigate whether dose dependence existed. Mice were treated for 1 week, 4 weeks, and 12 weeks to systematically explore the acute, subacute, and subchronic toxicity of PS-NPs. Finally, high-throughput sequencing was performed to probe the potential mechanisms of cardiac toxicity induced by PS-NPs after different exposure periods. The present study may help to provide new insights into the cardiotoxicity of PS-NPs and act as a wake-up call to raise awareness of protecting environment.

## Results

### Characterization of PS-NPs

**Fig. 1:**
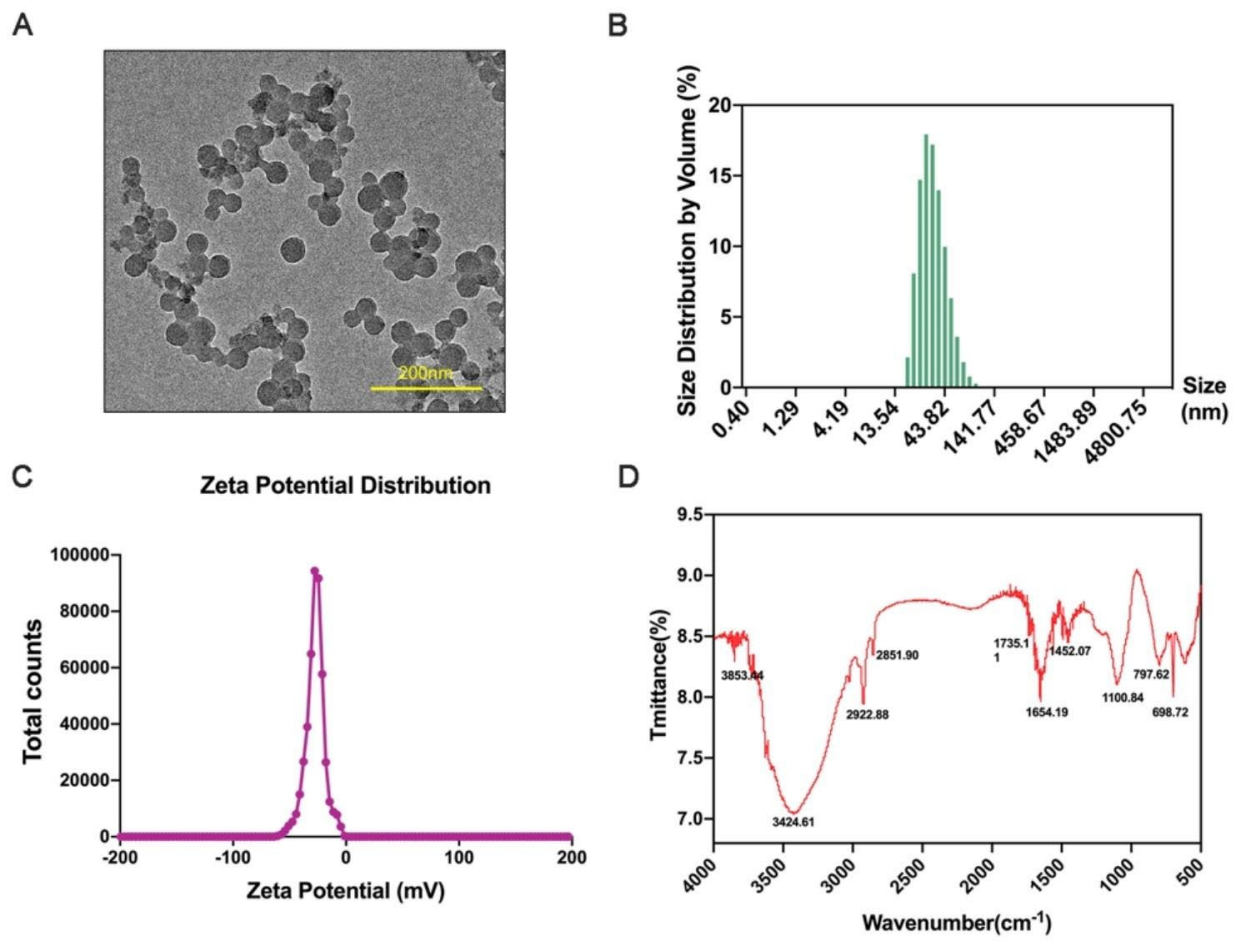
Characterization of PS-NPs. (**A**): TEM images of 40 nm PS-NPs (scale bar: 200 nm). (**B**): Size distribution by volume. (**C**): Zeta potential distribution of PS-NPs. (**D**): FTIR spectroscopy of PS-NPs

Transmission electron microscopy (TEM) was used to measure the shape and size of PS-NPs. As shown in Fig. 1A, PS-NPs showed a round shape. Dynamic light scattering (DLS) was further performed to analyze the diameter of PS-NPs, and the exact particle size was 40.24 ± 16.36 nm (Fig. 1B). According to FTIR analysis, the chemical composition was polystyrene (Fig. 1C). The zeta potential value of PS-NPs was − 27.8 ± 8.28 mV (Fig. 1D).

### Accumulation of PS-NPs in the heart and the effects on mouse body weight, heart weight and heart/body weight index after respiratory exposure to PS-NPs

**Fig. 2:**
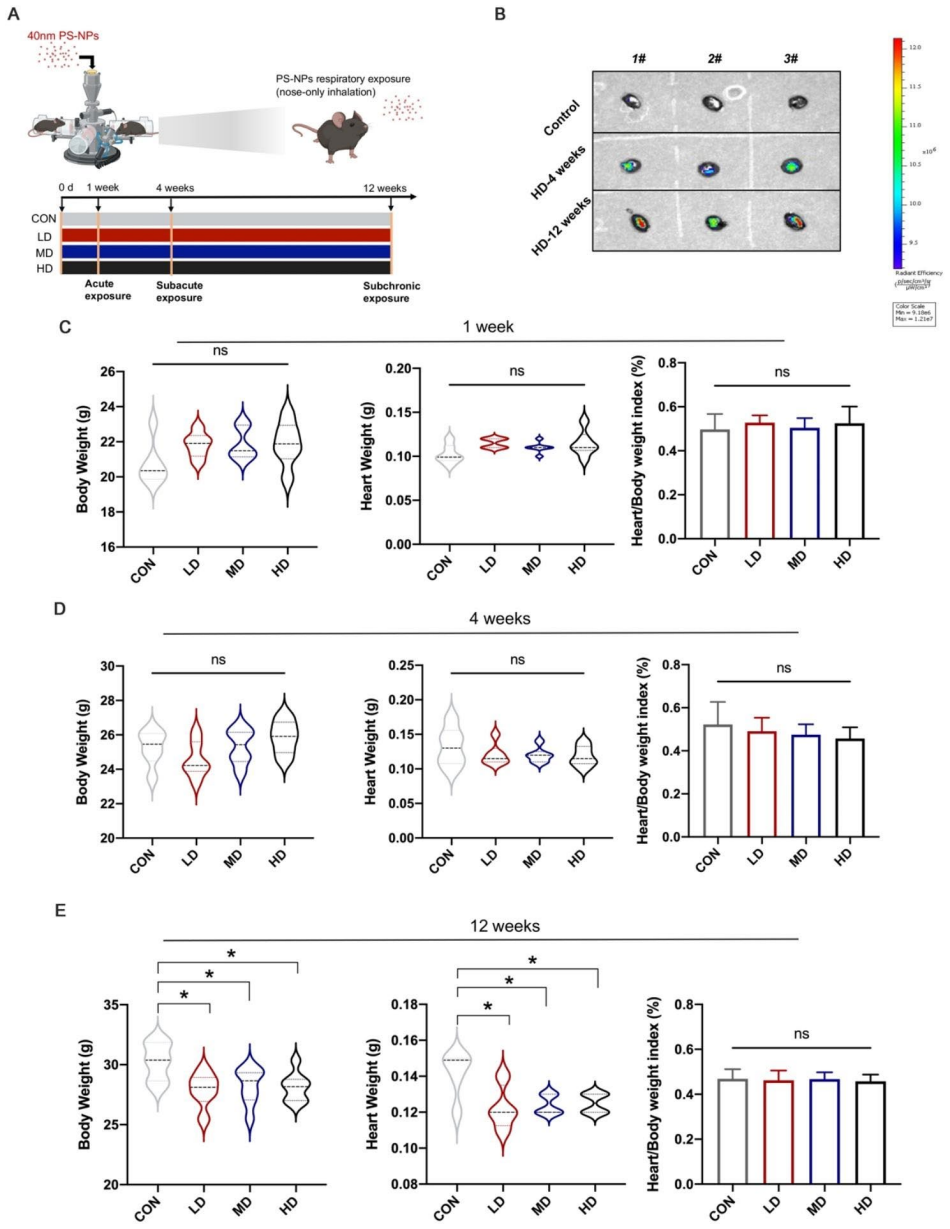
Accumulation of PS-NPs in the heart and the effects on mouse body weight, heart weight and heart/body weight index after respiratory exposure to PS-NPs. (**A**): Scheme of the experiment. (**B**): Representative IVIS images of mice in the control group and HD groups after 4 weeks of exposure and 12 weeks of exposure. Body weight, heart weight and heart/body weight index of mice were detected after PS-NPs exposure for 1 week (**C**) 4 weeks (**D**) and 12 weeks (**E**). Bar graphs indicate the mean ± SD. *P < 0.05

Six-week-old C57BL/6 mice were exposed to low-dose (LD), medium-dose (MD) and high-dose (HD) fluorescent PS-NPs consecutively for 1 week, 4 weeks, and 12 weeks, respectively, via inhalation (Fig. 2A). HD groups were selected to determine the distribution of fluorescent PS-NPs in mice. IVIS imaging showed that PS-NPs aggregated in the chest and abdomen of mice after exposure for 4 weeks and 12 weeks (Figure S2). To further clarify the accumulation of PS-NPs in the heart, we removed the hearts for imaging. Compared to the control group, the cardiac fluorescence signal was stronger in the 4-week and 12-week groups, and it appeared that more PS-NPs aggregated in the heart as the exposure duration increased (Fig. 2B). We further detected the effects of respiratory exposure to PS-NPs on mouse body weights, heart weights and heart/body weight index. There were no changes in body weights, heart weights, or heart organ coefficients after 1 week and 4 weeks of exposure (Fig. 2C–D). However, twelve weeks of exposure resulted in significant body and heart weight loss in mice, with no change in the heart/body weight index (Fig. 2E).

### Effects of PS-NPs exposure on heart function and structure

**Fig. 3:**
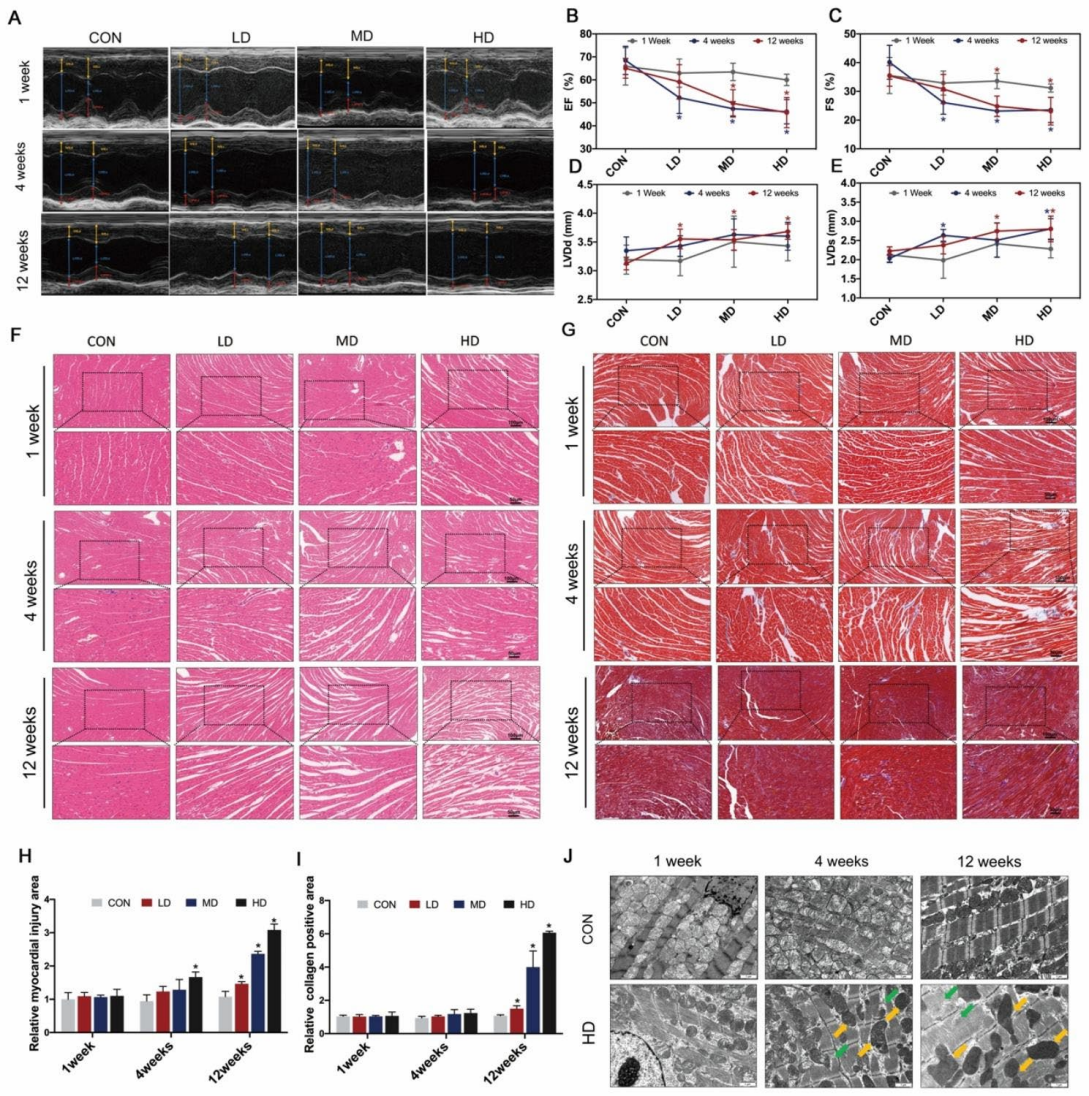
Effects of PS-NPs exposure on heart function and structure. (**A**): Representative M-mode echocardiograms. Yellow line with diamond arrow represented IVS; d. Yellow line with triangle arrow represented IVS; s. Blue line with diamond arrow represented LVID; d. Blue line with triangle arrow represented LVID; s. Red line with diamond arrow represented LVPW; d. Red line with triangle arrow represented LVPW; s The mean changes in EF (**B**), FS (**C**), LVDd (**D**) and LVDs (**E**) were displayed. (**F**–**G**): HE staining and Masson staining (100 × and 200 ×) of heart tissues. (**H**–**I**): Quantification of injury area and fibrosis levels. (**J**): Subcellular structure of the myocardium by TEM. Green arrow: fractured myofilaments. Yellow arrow: damaged mitochondria. Bar graphs indicate the mean ± SD. *P < 0.05. IVS; d: Interventricular septal thickness at diastole; IVS; s: Interventricular septal thickness at systole; LVID; d (LVDd for short): Left ventricular cavity diameter at diastole; LVID; s (LVDs for short): Left ventricular cavity diameter at systole; LVPW; d: Left ventricular posterior wall thickness at end-diastolic; LVPW; s: Left ventricular posterior wall thickness at end-systolic. EF: ejection fraction; FS: fractional shortening. Bar graphs indicate the mean ± SD. *P < 0.05

We used echocardiography to evaluate the cardiac performance of mice after exposure to PS-NPs (Fig. 3A). There was no change in ejection fraction (EF) or short axis fractional shortening (FS) after 1-week exposure. However, the values of EF and FS significantly decreased in a dose-dependent manner after 4 weeks and 12 weeks of exposure. Notably, mice exposed to LD and MD for 4 weeks showed lower EF and FS values than the same dose group exposed for 12 weeks (Fig. 3B–C). Additionally, after treatment with PS-NPs, the serrated profile of the Doppler blood flow spectrum became flatter, and LVDd and LVDs increased significantly, which indicated signs of left ventricular concentric remodeling (Fig. 3A, D–E). To further assess the changes in cardiac structure, HE staining was performed. As shown in Fig. 3F, the myocardium was arranged neatly and tightly in the control group, whereas disorganized myofilament arrangement and myocardial fragmentation started to appear in the HD group after exposure for 4 weeks. More seriously, even the LD group exhibited significant cardiac structural damage after 12-week exposure, and the myocardial injury area expanded as the exposure dose increased (Fig. 3F, H). Since persistent myocardial damage frequently results in cardiac fibrosis, we used Masson staining to measure the degree of collagen deposition. The level of cardiac fibrosis was considerably increased after exposure for 12 weeks, whereas no change was found in the 4-week group compared to the control group (Fig. 3G, I). We used TEM to detect ultrastructural damage in the heart. Compared to the control group, the myocardial fibers were disordered and disrupted, with mitochondria swelling and deforming in the HD group after 4 weeks of exposure. Moreover, the cristae of mitochondria disappeared gradually, and the above ultrastructural damage was more obvious as the exposure time was prolonged to 12 weeks (Fig. 3J).

### Effects of PS-NPs exposure on the levels of myocardial injury biomarkers

**Fig. 4:**
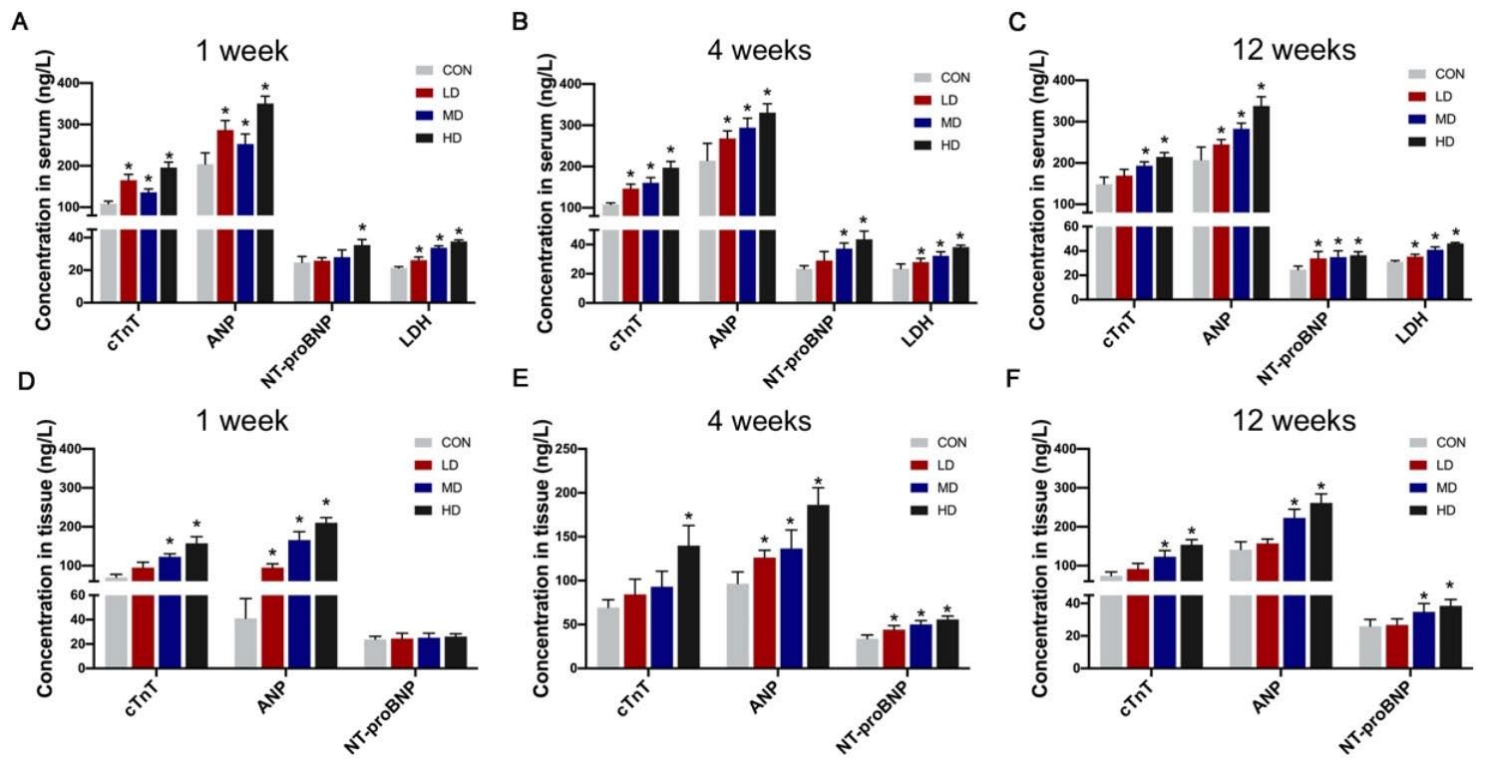
Effects of PS-NPs exposure on the levels of myocardial injury biomarkers. The levels of cTnT, ANP, NT-proBNP and LDH in serum after mice were exposed to PS-NPs for 1 week (**A**), 4 weeks (**B**) and 12 weeks (**C**). The levels of cTnT, ANP, and NT-proBNP in heart tissues after exposure for 1 week (**D**), 4 weeks (**E**) and 12 weeks (**F**). Bar graphs indicate the mean ± SD. *P < 0.05

We then detected the levels of cardiac biomarkers such as cardiac Troponin T (cTnT), ANP, N-terminal proBNP (NT-proBNP) and LDH in serum and cardiac tissues. The concentration of the biomarkers released in the serum significantly increased after PS-NPs exposure for only 1 week and remained high in the 4-week and 12-week exposure groups (Fig. 4A–C). As the PS-NPs exposure dose increased, the content of injury biomarkers in serum was steadily rising. Moreover, similar trends were also observed in cardiac tissues (Fig. 4D–F).

### Effects of PS-NPs exposure on the inflammatory response and oxidative stress levels

**Fig. 5:**
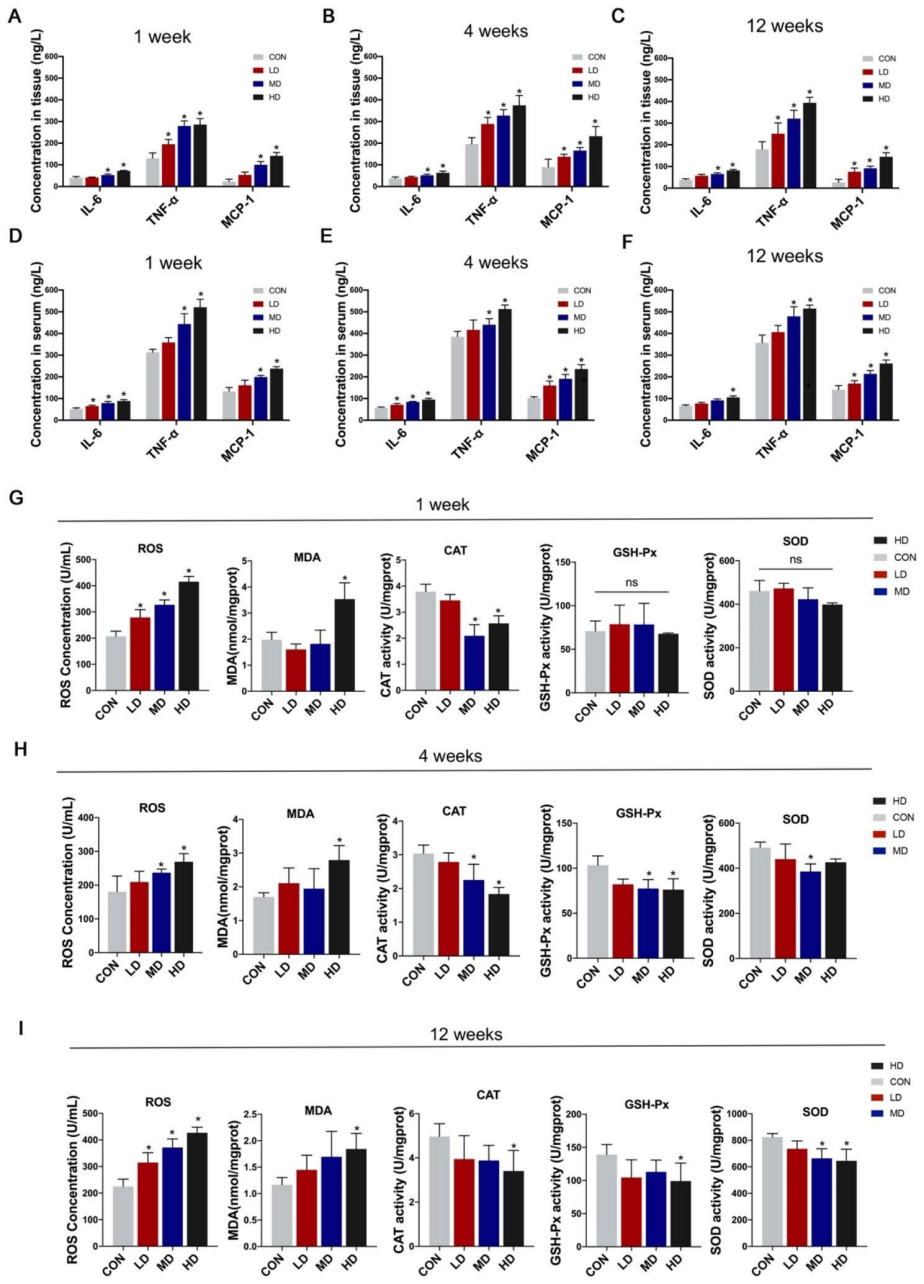
Effects of PS-NPs exposure on the inflammatory response and oxidative stress levels. The concentrations of IL-6, MCP-1 and TNF-α in heart tissues after PS-NPs exposure for 1 week (**A**), 4 weeks (**B**) and 12 weeks (**C**) and the concentrations in serum after PS-NPs exposure for 1 week (**D**), 4 weeks (**E**) and 12 weeks (**F**). The concentrations of ROS, MDA, CAT, GSH-Px and SOD in heart tissues after PS-NPs treatment for 1 week (**G**), 4 weeks (**H**) and 12 weeks (**I**). Bar graphs indicate the mean ± SD. *P < 0.05

PS-NPs dramatically activated the inflammatory response in heart tissues among all exposure duration groups (Fig. 5A–C). The levels of inflammation in serum were also significantly elevated in a dose-dependent manner compared to the corresponding control groups (Fig. 5D–F). We further assessed the levels of oxidative stress in cardiac tissues. The 1-week, 4-week, and 12-week exposure groups all experienced a rise in reactive oxygen species (ROS) levels (Fig. 5G–I). Since the increase in oxidative stress levels was caused by a disruption in the equilibrium between oxidase and antioxidase enzyme activity, we detected the content of malondialdehyde (MDA) and the activity of catalase (CAT), glutathione peroxidase (GSH-Px) and superoxide dismutase (SOD). Our findings demonstrated that following high-dose PS-NPs treatment, MDA content markedly increased in the three exposure duration groups, whereas CAT activity trended downward (Fig. 5G–I). Furthermore, compared to the control groups, GSH-Px and SOD activity were stable in the 1-week group but declined after 4 and 12 weeks of PS-NPs exposure (Fig. 5G–I).

### Transcriptome profiling of mouse heart tissues in response to PS-NPs

**Fig. 6:**
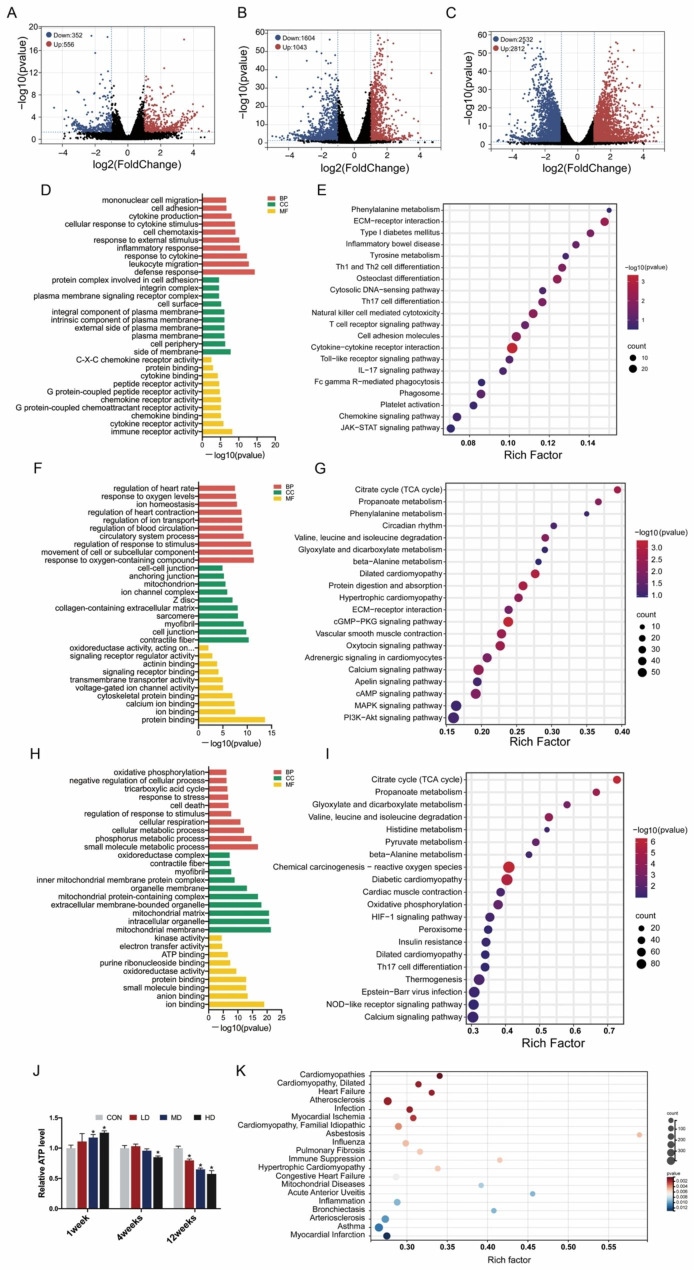
Transcriptome profiling of mouse heart tissues in response to PS-NPs after 1 week, 4 weeks and 12 weeks. Three mice in the HD groups and corresponding control groups were randomly selected for RNA-seq after PS-NPs treatment for 1 week, 4 weeks and 12 weeks. Volcano plots of DEGs were depicted by comparison between the CON and HD groups at 1 week (**A**), 4 weeks (**B**) and 12 weeks (**C**). GO terms and KEGG pathways were enriched based on the DEGs from the 1-week exposure group (**D**–**E**), 4-week exposure group (**F**–**G**) and 12-week exposure group (**H**–**I**). ATP content in heart tissues was evaluated after exposure for 1 week, 4 weeks and 12 weeks (**J**). The DEGs in the 12-week exposure group were selected to perform disease annotation analysis (**K**). Bar graphs indicate the mean ± SD. *P < 0.05

We employed RNA-seq to explore the changes in transcriptome profiles after PS-NPs treatment for 1 week, 4 weeks and 12 weeks. The heart samples of the HD groups in three exposure durations were selected for comparison with the corresponding control groups due to the most significant cardiac injury. As the exposure period was prolonged, the number of differentially expressed genes (DEGs) increased significantly. There were 908 genes markedly changed in the 1-week exposure group, with 556 genes upregulated and 352 genes downregulated (Fig. 6A). Moreover, 4 weeks of treatment led to 2647 DEGs, of which 1043 genes were upregulated and 1604 genes were downregulated (Fig. 6B). After exposure for 12 weeks, the number of DEGs jumped to 5344, including 2812 upregulated genes and 2532 downregulated genes (Fig. 6C).

To better understand these DEGs, KEGG and GO annotation analysis were performed. We displayed 30 most significant GO terms classified by “Biological Process (BP)”, “Cellular Component (CC)” and “Molecular Function (MF)” and the top 20 enriched KEGG pathways for each pair of comparison groups. After exposure to PS-NPs for 1 week, the terms “defense response”, “leukocyte migration”, “response to cytokine”, “inflammatory response” and “response to external stimulus”, etc., were significantly enriched (Fig. 6D), and pathways such as “phenylalanine metabolism” and “ECM-receptor interaction” were dramatically activated (Fig. 6E). In the 4-week treatment group, the terms “protein binding”, “response to oxygen-containing compound”, “movement of cell or subcellular component”, “regulation of response to stimulus” and “contractile fiber” were ranked in the top 5 (Fig. 6F). The pathways “citrate cycle (TCA cycle)” and “propanoate metabolism” got the highest and second highest rich factors, respectively, which was the same in the 12-week exposure group (Fig. 6G, I). In addition, mitochondria-related terms were remarkedly enriched in the 12-week exposure group, including “mitochondrial membrane”, “mitochondrial matrix”, “mitochondrial protein-containing complex”, “inner mitochondrial membrane protein complex”, etc. (Fig. 6H). ATP content was further tested to evaluate mitochondrial function and it significantly decreased after 12-week exposure (Fig. 6J), which indicated mitochondrial damage may play a pivotal role in cardiac injury after prolonged exposure.

To investigate the potential diseases induced by long-term PS-NPs treatment, we selected the DEGs in the 12-week exposure group to perform disease annotation analysis. As shown in Fig. 6K, numerous cardiovascular diseases were enriched, such as “Cardiomyopathies”, “Cardiomyopathy, Dilated”, “Heart Failure” and “Atherosclerosis”, which revealed the cardiovascular toxicity of PS-NPs respiratory exposure. In addition, respiratory diseases, e.g., “Pulmonary Fibrosis”, “Bronchiectasis”, and “Asthma” are also highly susceptible to PS-NPs exposure.

## Discussion

The environmental burden caused by plastics has become a global problem. Due to their small particle size, MNPs are capable of entering the blood system and accumulating in multiple organs of human body, causing potential toxicity [24, 25]. Notably, the heart has recently been found to be a target organ accumulated and damaged by MNPs [17, 18, 26]. In addition, as the study develops in depth, respiratory intake was found to be an inevitable way for MNPs contact in daily life [27, 28]. Due to the special physiological structure of the cardiopulmonary system, theoretically, MNPs could flow into the left ventricle through pulmonary veins after penetrating the air‒blood barrier, thus, bringing out potential cardiac damage by direct cytotoxicity. On the other hand, a growing body of research has confirmed that respiratory exposure to MNPs disrupts the oxidative balance of the pulmonary system and increases oxidative stress and inflammation levels in the lungs and blood circulation, which may result in the cardiovascular system being constantly stimulated by risk factors and subsequently increasing potential heart damage [29–32]. Regrettably, the current research on MNPs-induced cardiac injury remains insufficient, and in almost all relevant studies, MNPs were administered orally [18]. Therefore, we adopted respiratory exposure to PS-NPs in this study, and multiple dosage level treatment were designed according to the actual environmental exposure concentrations. Moreover, multiple exposure periods were arranged to observe short-term and long-term effects on cardiac injury, and the underlying mechanisms were revealed by high-throughput sequencing. To the best of our knowledge, this study was the first to systematically demonstrate cardiotoxicity induced by PS-NPs respiratory exposure for acute, subacute and subchronic periods.

Our study showed that the cardiac fluorescence signal was significantly enhanced after 4 weeks of respiratory exposure to fluorescent PS-NPs, and the intensity further increased as the exposure period was prolonged to 12 weeks. This result suggested that PS-NPs could accumulate in the heart, which was consistent with previous findings [21]. Echocardiography could provide a noninvasive view of cardiac structure and function, and it showed that exposure to PS-NPs for 4 weeks and 12 weeks dose-dependently decreased cardiac EF and FS, which indicated impairment in cardiac systolic function. LVIDs and LVIDd reflected the inner diameter of the left ventricle at systole and diastole, the increase of which implied that mice gained an enlarged left ventricular cavity after subacute and subchronic exposure. This was consistent with the results of our disease annotation analysis (“Cardiomyopathy, Dilated”). According to the results of HE staining, the HD group after 4-week exposure showed pathological arrangement, whereas all the groups after 12 weeks of exposure manifested obvious cardiac structural damage, which demonstrated that the long-term exposure risk of PS-NPs cannot be ignored, even at relatively low concentrations. Fibrosis, as a defensive response to acute cardiac injury, contributes to stabilizing the heart and maintaining integrity and normal function to a certain extent [33]. However, excessive fibrosis reduces cardiac compliance and accelerates the progression of heart failure [34–36]. Our results observed that there was no obvious fibrosis after 1 week and 4 weeks of exposure. However, the degree of fibrosis increased in a dose-dependent manner after PS-NPs exposure for 12 weeks. Interestingly, based on the echocardiography results, after treatment with LD and MD PS-NPs for 12 weeks, the cardiac systolic function of mice was better than that of mice exposed for 4 weeks. We speculated that the mice in the 12-week exposure groups developed heart compensatory behaviors, e.g., cardiac hypertrophy, and maintained cardiac function partly due to the function of fibrosis in stabilizing the heart after acute injury. A previous report noted that MPs exposure led to lesions in the subcellular structure of the myocardium [20, 21]. The current study observed significant damage to mitochondria and myofilaments after 4 weeks and 12 weeks of treatment. Mitochondria occupies a central position in the biology of the myocardium due to their major role in energy metabolism and regulation of ion homeostasis [37]. Mitochondrial defects have been found to participate in the pathogenesis of a variety of cardiovascular disorders [38]. In our study, mitochondrial damage was positively correlated with cardiac dysfunction and structural damage, which preliminarily provided some clues for the potential mechanisms of PS-NPs-induced cardiac damage.

Compared to the functional loss and structural disorders in the heart, cardiac biomarkers are more sensitive and could reflect myocardial injury in the early stage, so we detected biomarkers, including cTnT, ANP, NT-proBNP and LDH. Notably, although obvious damage was not observed by echocardiography and HE staining until PS-NPs treatment for 4 weeks, the levels of cTnT, ANP and LDH were significantly elevated after only 1-week exposure, suggesting that PS-NPs exposure induced acute cardiotoxicity.

Our study observed that inflammation responded rapidly to cellular damage, with 1-week PS-NPs exposure significantly increasing the concentrations of inflammatory factors in cardiac tissues. With continuous PS-NPs treatment, inflammation levels remained stubbornly high in heart tissues. Notably, our study revealed that respiratory exposure to PS-NPs also induced elevated levels of systemic inflammation, which may be attributed to potential multiorgan toxicity. Numerous studies have regarded high levels of systemic inflammation as a risk factor for cardiovascular disease [39–42], so the cardiac injury caused by PS-NPs inhalation may be a compound effect mediated by the direct cardiotoxicity caused by PS-NPs accumulation in the heart and the indirect toxicity caused by elevated systemic inflammation levels. It has been reported that triggering oxidative stress is one of the typical toxic effects induced by nanoparticles and participates in MNPs-induced cardiotoxicity [20, 43]. The production of ROS could activate a plethora of signaling cascades, e.g., p53 signaling pathway, Mitogen-activated protein kinases (MAPKs), etc., resulting in organ damage [32]. In our study, ROS contents in heart tissues were maintained at high levels throughout all three exposure periods, which was attributed to the imbalance of oxidase and antioxidant enzyme activities.

Mechanistically, with a prolonged exposure period, PS-NPs induced more widespread changes in the cardiac transcriptome, reflected in a significantly higher number of DEGs. Based on GO and KEGG analysis, acute exposure activated strong inflammatory and immune responses in mouse hearts, as shown by “leukocyte migration”, “response to cytokine”, “inflammatory response”, etc., from GO annotation and “cell adhesion molecules”, “chemokine signaling pathway”, “cytokine‒cytokine receptor interaction”, etc., from KEGG pathway analysis. Following subacute exposure, we noted that ion transport, cardiac contraction and mitochondrial function appeared to be interfered with by PS-NPs, according to the GO terms “regulation of ion transport”, “contractile fiber”, “ion binding”, “voltage-gated ion channel activity”, “mitochondrion”, etc. KEGG pathway analysis showed that “cardiac rhythm” and “calcium signaling pathway” were significantly enriched, which corroborated the GO analysis. Notably, based on KEGG analysis, subacute exposure could disturb the energy metabolism of cardiomyocytes, of which the “TCA cycle” term was enriched most significantly. Similarly, subchronic treatment of PS-NPs would interfere with mitochondrial structure and function, ion transport, cardiac contraction, and energy metabolism. However, much more mitochondria-related terms were enriched, suggesting that consecutive PS-NPs exposure may aggravate mitochondrial damage, which was consistent with our TEM observations. It was worth mentioning that the “TCA cycle” term was also most significantly enriched. Cardiomyocytes have the highest abundance of mitochondria due to their heavy reliance on adenosine triphosphate (ATP) [44, 45]. The TCA cycle contributes to generating ATP by oxidizing acetyl-CoA, derived from sugar, fat and amino acids, which is essential for energy supply. Thus, we boldly speculated that metabolic disturbances, especially in the TCA cycle, caused by mitochondrial damage may be an important mechanism of cardiotoxicity induced by subacute and subchronic PS-NPs exposure. The results of the ATP content test have partially confirmed our hypothesis, and more detailed experiments of mitochondrial function evaluation and energy metabolism detection needs to be carried out in the future. For sure, clarifying the time-coursed linkage of cardiac contraction forces, calcium ion transport, mitochondrial membrane potentials, reactive oxygen species levels, etc., would enable us to build up the sequence of the cellular events associated with the detrimental effects of PS-NPs on cardiomyocytes, as described by Amir et al. [46].

Despite the advances proffered by our study, major limitations exist that dampen enthusiasm. First, we have already detected the accumulation of PS-NPs in mouse hearts via in vivo imaging; however, we were unable to quantitatively measure its concrete content due to the limitation of detection technology for NPs. In addition, as shown by the serum ELISA results, respiratory exposure to PS-NPs caused elevated levels of systemic inflammation, which may indirectly lead to cardiac injury. The extent to which the cardiac injury of mice is attributable to the direct cardiomyocyte toxicity of PS-NPs needs to be further clarified. Moreover, high-throughput sequencing analysis provided preliminary clues about the potential mechanisms, and it needs to be explored more deeply. Finally, exposure of PS-NPs to human body in the real ambient environment would be more complex, and as reveled by Lin et al. [26], the cardiotoxicity induced by combined effects of PS-NPs exposure with other pollutants needs to be given more attention in the further study.

## Conclusion

Overall, this study systematically investigated cardiac injury in mice induced by multi-concentration and multi-period respiratory exposure to PS-NPs. The results showed that PS-NPs exposure induced acute cardiotoxicity in a dose-dependent manner. Subacute and subchronic treatment resulted in the accumulation of PS-NPs in the heart, with more structurally and functionally damaging effects than acute exposure. Long-term exposure to PS-NPs may lead to potential cardiovascular diseases, which may be attributed to damaged mitochondria according to transcriptome sequencing analysis. The existing limitations need to be overcome to further explore the mechanisms of PS-NPs-induced cardiotoxicity.

## Materials and methods

### Characterization of PS-NPs

Fluorescent unmodified polystyrene nanoparticles (PS-NPs, 40 nm, 50 mg/ml) were purchased from Huge Biotechnology Corporation (Shanghai, China). Transmission electron microscopy (TEM, FEI, F20, America) and Fourier transform infrared spectroscopy (FTIR) (Thermo Fisher Scientific, USA) were used to identify the morphology and composition of PS-NPs. Zetasizer Nano-ZS90 instrument (Malvern Instruments, Malvern, UK) was used to measure the hydrodynamic diameter and zeta-potential of PS-NPs in 0.9% saline.

### Animals and treatment

Male C57BL/6J mice (6 − 8 weeks, 18–22 g) were purchased from Beijing Vital River Laboratory Animal Technology and housed in a specific pathogen-free (SPF) environment with temperatures of 22 ± 2 °C, humidity levels of 50–60% and an equal light-dark cycle. The study was approved by the Institutional Animal Care and Use Committee (IACUC) of Southeast University. Mice were randomly separated into a total of 12 groups (n = 6 per group) including four exposure dosage levels (control group (CON, 0.9% saline), low dose group (LD), medium dose group (MD), high dose group (HD)) and three exposure periods (1 week, 4 weeks and 12 weeks). According to the latest report on the measurement of microplastic content in the real situation, the average microplastic concentrations in the air in China and the world have reached 282 [47] and 638 items/m^3^ [48], respectively. Moreover, the concentration of indoor microplastics even reached 1583 items/m^3^. Therefore, we chose these three known doses as the basis to set the low-, medium- and high-dose groups. Due to the limitation of nanoparticle detection technology, we mathematically converted atmospheric microparticles with a diameter of 100 μm to our 40 nm polystyrene microspheres, as described previously [27, 49]. A single microparticle represented 2.9 × 10^10^ 40 nm nanoparticles. Thus, according to our conversion, the concentration of nanoplastics exposed in the air may be 7.9 × 10^12^ 1.85 × 10^13^ and 4.6 × 10^13^ items/m^3^, corresponding to low, medium and high dosages, respectively. Due to differences in body weight and body surface area between humans and mice, the normal respiratory volume of mice and extrapolation coefficient were taken into account. The dose settings complied with the realistic situation and were calculated by the following formula, as previously described [50]: Dose of PS-NPs exposure = Realistic PS-NPs concentration × Respiratory volume × Exposure time × Extrapolation coefficient. Combined with the studies of Wu et al. [27], the appropriate doses were ultimately set as 16 µg/day (LD), 40 µg/day (MD) and 100 µg/day (HD) for each mouse.

DSI Buxco Inhalation Exposure System(Data Sciences International [DSI] St. Paul, MN) was used for PS-NPs respiratory exposure (Figure S1). Different doses of PS-NPs were suspended in 0.9% saline and fed into the nebulizer. Mice were gently placed into the chambers and connected to the inhalation tower. Before exposure, Finepoint software (Data Sciences International [DSI] St. Paul, MN) was used to detect the respiratory rate of mice. The PS-NPs suspension was nebulized after the breathing rhythm was steady. Respiratory parameters, including breaths per minute, tidal volume, etc., as well as parameters of the inhalation tower, such as flow, pressure and humidity, were monitored in real time. All dosage groups were exposed to PS-NPs for 1 week, 4 weeks and 12 weeks. After the last treatment, all mice were gently sacrificed for the following experiments.

### Fluorescence imaging in vivo and ex vivo

Mice in the high-dose group were selected to examine the bioaccumulation of PS-NPs in mouse hearts after treatment for 4 weeks and 12 weeks. Isoflurane was adopted for anesthetization, and hair on the chest and abdomen of the mice was shaved to track the fluorescent signal in vivo more clearly. After that, heart tissues were collected for in vitro fluorescence imaging to exclude the interference of fluorescent signals in the lungs. An IVIS Spectrum system (PerkinElmer, USA) was used to obtain fluorescence images with a 450 nm excitation filter and 680 nm emission filter. Living Image® software (PerkinElmer, USA) was used for image processing.

### In vivo cardiac functional assessment

The left ventricular morphology and left ventricular systolic function parameters were detected by echocardiography (Vevo 3100, Fujifilm Visualsonics Inc., Japan). Depilatory ointment was applied to the chest of the mouse to remove the hair, and 1% isoflurane was used for anesthetization. Mice were fixed supine on a 37 °C constant-temperature operating table for two-dimensional and M-mode echocardiographic image acquisition. The following parameters were recorded: left ventricular cavity diameter at systolic (LVDs), left ventricular cavity diameter at diastole (LVDd), left ventricular posterior wall thickness at end-diastole (LVPWd), left ventricular posterior wall thickness at end-systole (LVPWs), interventricular septum diameter at diastole (IVSd), interventricular septum diameter at systole (IVSs), ejection fraction (EF) and fractional shortening (FS).

### Histopathological observation of the heart

The heart tissues were resected and fixed immediately in 4% paraformaldehyde for 4 h. After being dehydrated with a graded ethanol series, the embedded tissues were cut into 5 μm sections and then stained by hematoxylin and eosin (HE staining) and Masson’s trichrome staining. Pictures were taken using a microscope (Olympus IX83, Japan). Quantifications were performed with ImageJ. For each mouse, three slides were stained, and five fields in each section were collected randomly and analyzed.

### Transmission electron microscopy (TEM) observation

Approximately 1 mm^3^ sections of heart samples were harvested and fixed in 3% glutaraldehyde for 2 h, followed by 2% OsO4 for 2 h. After dehydration in gradient ethanol, all the samples were embedded in resin and then cut into 70 nm ultrathin sections. The sections were stained with uranyl acetate and lead citrate and observed by transmission electron microscope (Hitachi Model H-7650, Tokyo, Japan).

### Detection of cardiac injury biomarkers and inflammation

Heart tissues and serum of mice were collected to evaluate the levels of cardiac injury biomarkers and inflammation. According to the manufacturer’s instructions, the contents of cardiac Troponin T (cTnT), ANP, N-terminal proBNP (NT-proBNP), LDH, IL-6, MCP-1 and TNF-α were qualified by ELISA kits (YIFEIXUE Biotechnology, Nanjing, China).

### Oxidative stress evaluation

The levels of reactive oxygen species (ROS) in heart tissues were measured by ELISA kits (YIFEIXUE Biotechnology, Nanjing, China). The content of superoxide dismutase (SOD) and the enzyme activities of catalase (CAT), glutathione peroxidase (GSH-Px), and malondialdehyde (MDA) were measured by commercial kits following the manufacturer’s instructions (Jiancheng Bioengineering Institute, Nanjing, China).

### RNA sequencing and data analysis

After exposure to PS-NPs for 1 week, 4 weeks, and 12 weeks, three heart samples were selected from the high-dose group and the corresponding control group for RNA-seq at each exposure time point. RNA-seq was performed as previously described [51]. Total RNA was extracted from heart tissues using TRIzol reagent according to the manufacturer’s protocol. A NanoDrop 2000 spectrophotometer (Thermo Scientific, USA) was used to evaluate RNA purity and quantification. RNA integrity was assessed using the Agilent 2100 Bioanalyzer (Agilent Technologies, Santa Clara, CA, USA). Then, the libraries were constructed using the TruSeq Stranded mRNA LT Sample Prep Kit (Illumina, San Diego, CA, USA) according to the manufacturer’s instructions and sequenced on an Illumina HiSeq X Ten platform, and 150 bp paired-end reads were generated. Raw data (raw reads) in fastq format were first processed using Trimmomatic, and low-quality reads were removed to obtain clean reads. The clean reads were mapped to the mouse genome (Mus_musculus. GRCm 38.99) using HISAT2. The FPKM of each gene was calculated using Cufflinks, and the read counts of each gene were obtained by HTSeqcount. Differential expression analysis was performed using the DESeq (2012) R package and is shown as volcano plots. P value < 0.05 and fold change ≥ 2 or ≤ 0.5 were set as the thresholds for significantly differential expression. GO enrichment and KEGG pathway enrichment analysis of DEGs were performed using R based on the hypergeometric distribution. Disease enrichment analysis was performed based on the DisGeNET database (https://www.disgenet.org).

### Evaluation of mitochondria function

ATP content detection was used to evaluate mitochondria function. Cardiac tissues of mice were collected and the concentrations of ATP were measured by commercial kits following the manufacturer’s instructions (Beyotime, Beijing, China).

### Statistical analysis

One-way ANOVA with Dunnett’s multiple comparison test was performed by SPSS software (IBM SPSS v18.0, Chicago, IL, USA). P values < 0.05 were considered statistically significant (*).

### Electronic supplementary material

Below is the link to the electronic supplementary material.

Supplementary Material 1: DSI Buxco Inhalation Exposure System. (A): Design pattern diagram of DSI Buxco Inhalation Exposure System. (B): Inhalation tower. (C): Chamber of Inhalation Exposure. (D): All-in-one controller of Buxco Inhalation tower. (E): Finepoint software. Supplementary figure 2: Accumulation of PS-NPs in the heart and the effects on mice body weight, heart weight and heart/body weight index after respiratory exposure to PS-NPs. IVIS images of mice in vivo for the control group (A), HD groups after 4-weeks exposure (B) and 12-weeks exposure (C).

## Data Availability

The datasets used and/or analysed during the current study are available from the corresponding author on reasonable request.

## Abbreviations

NPs: Nanoplastics
PS-NPs: Polystyrene nanoplastics
MPs: Microplastics
MNPs: Micro(nano) plastics
BALF: Bronchoalveolar lavage fluid
TEM: Transmission electron microscopy
DLS: Dynamic light scattering
EF: Ejection fraction
FS: Short axis fractional shortening
LVDd: Left ventricular cavity diameter at diastole
LVDs: Left ventricular cavity diameter at systolic
LVPWd: Left ventricular posterior wall thickness at end-diastole
LVPWs: Left ventricular posterior wall thickness at end-systole
IVSd: Interventricular septum diameter at diastole
IVSs: Interventricular septum diameter at systole
cTnT: Cardiac Troponin T
NT-proBNP: N-terminal proBNP
MDA: Malondialdehyde
CAT: Catalase
GSH-Px: Glutathione peroxidase
SOD: Superoxide dismutase
ATP: Adenosine triphosphate
