# Supplementary material for: Multi-dimensional evaluation of cardiotoxicity in mice following respiratory exposure to polystyrene nanoplastics
Source: Part Fibre Toxicol. 2023 Nov 29;20:46. doi: 10.1186/s12989-023-00557-3 (PMC10685678; doi:10.1186/s12989-023-00557-3)
Supplement: Supplementary file 1 — Supplementary Material 1: DSI Buxco Inhalation Exposure System. (A): Design pattern diagram of DSI Buxco Inhalation Exposure System. (B): Inhalation tower. (C): Chamber of Inhalation Exposure. (D): All-in-one controller of Buxco Inhalation tower. (E): Finepoint software. Supplementary figure 2: Accumulation of PS-NPs in the heart and the effects on mice body weight, heart weight and heart/body weight index after respiratory exposure to PS-NPs. IVIS images of mice in vivo for the control group (A), HD groups after 4-weeks exposure (B) and 12-weeks exposure (C). [file 12989_2023_557_MOESM1_ESM.docx]

**Additional file 1**

**Multi-dimensional evaluation of cardiotoxicity in mice following respiratory exposure to polystyrene nanoplastics**

**Tianyi Zhang^a^, Sheng Yang^a^, Yiling Ge^a^, Xin Wan^a^, Yuxin Zhu^a^, Fei Yang^b^, Jie Li^a^, Saisai Gong^a^, Yanping Cheng^a^, Chengyu Hu^a^, Zaozao Chen^c^, Lihong Yin^a^, Yuepu Pu^a^, Geyu Liang^a*^**

^a^ Key Laboratory of Environmental Medicine Engineering, Ministry of Education, School of Public Health, Southeast University, Nanjing, Jiangsu, 210009, China.

^b^ Hunan Province Key Laboratory of Typical Environmental Pollution and Health Hazards, School of Public Health, Hengyang Medical School, University of South China, Hengyang, 421001, China. ^c^ State Key Laboratory of Bioelectronics, School of Biological Science and Medical Engineering,

Southeast University, Nanjing, Jiangsu, 210096, China.

*** Corresponding Author：**

Email: lianggeyu@163.com (G. Liang), Tel: +86 25 83272572, Fax: +86 25 83324322

**Supplementary figure 1：**DSI Buxco Inhalation Exposure System. (A): Design pattern diagram of DSI Buxco Inhalation Exposure System. (B): Inhalation tower. (C): Chamber of Inhalation Exposure. (D): All-in-one controller of Buxco Inhalation tower. (E): Finepoint software.


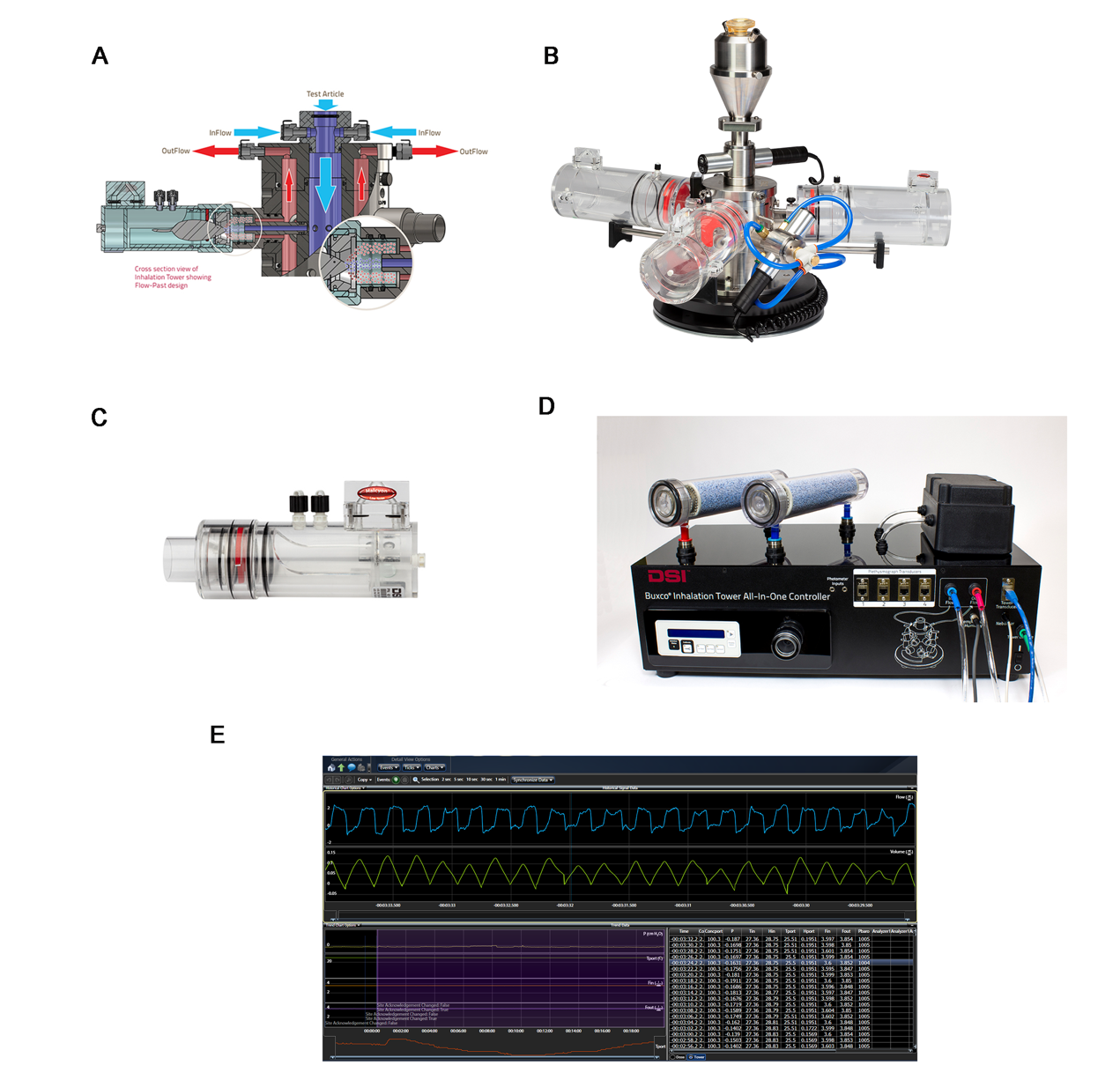


**Supplementary figure 2：Accumulation of PS-NPs in the heart and the effects on mice body weight, heart weight and heart/body weight index after respiratory exposure to PS-NPs.** IVIS images of mice in vivo for the control group (A), HD groups after 4-weeks exposure (B) and 12-weeks exposure (C).**
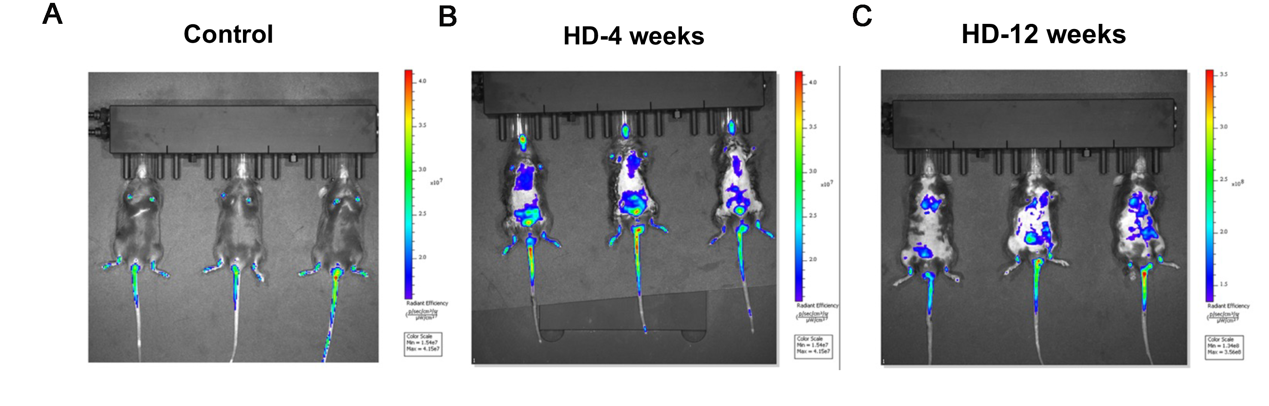
**
